# Intestinal Absorption of FITC-Dextrans and Macromolecular Model Drugs in the Rat Intestinal Instillation Model

**DOI:** 10.1021/acs.molpharmaceut.2c00261

**Published:** 2022-06-01

**Authors:** Staffan Berg, Denny Suljovic, Lillevi Kärrberg, Maria Englund, Heiko Bönisch, Ida Karlberg, Natalie Van Zuydam, Bertil Abrahamsson, Andreas Martin Hugerth, Nigel Davies, Christel A. S. Bergström

**Affiliations:** † The Swedish Drug Delivery Center, Department of Pharmacy, Uppsala University, BMC P.O. Box 580, Uppsala SE-751 23, Sweden; ‡ Advanced Drug Delivery, Pharmaceutical Sciences, R&D, AstraZeneca, Gothenburg 431 83, Sweden; § Animal Sciences and Technologies, Clinical Pharmacology and Safety Sciences, BioPharmaceuticals R&D, AstraZeneca, Gothenburg 431 83, Sweden; ∥ Drug Metabolism and Pharmacokinetics, Research and Early Development, Cardiovascular, Renal and Metabolism, BioPharmaceuticals R&D, AstraZeneca, Gothenburg 431 83, Sweden; ⊥ Affibody AB, Solna 171 65, Sweden; # Data Science and Quantitative Biology, Discovery Sciences, BioPharmaceuticals R&D, AstraZeneca, Gothenburg 431 83, Sweden; ∇ Oral Product Development, Pharmaceutical Technology & Development, Operations, AstraZeneca, Gothenburg 431 83, Sweden; ○ Ferring Pharmaceuticals A/S, Product Development and Drug Delivery, Global Pharmaceutical R&D, Amager Strandvej 405, Kastrup 2770, Denmark

**Keywords:** sodium caprate, permeation enhancer, FITC-dextran, MEDI7219, affibody molecule, oral peptide/protein delivery, rat intestinal instillation model

## Abstract

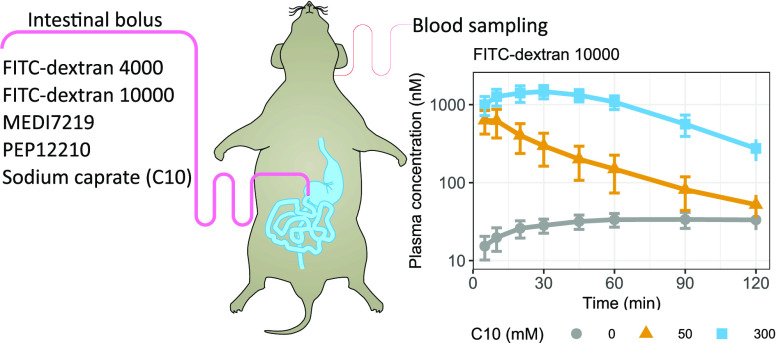

In this work, we studied the intestinal absorption of a peptide with a molecular weight of 4353 Da (MEDI7219) and a protein having a molecular weight of 11 740 Da (PEP12210) in the rat intestinal instillation model and compared their absorption to fluorescein isothiocyanate (FITC)-labeled dextrans of similar molecular weights (4 and 10 kDa). To increase the absorption of the compounds, the permeation enhancer sodium caprate (C10) was included in the liquid formulations at concentrations of 50 and 300 mM. All studied compounds displayed an increased absorption rate and extent when delivered together with 50 mM C10 as compared to control formulations not containing C10. The time period during which the macromolecules maintained an increased permeability through the intestinal epithelium was approximately 20 min for all studied compounds at 50 mM C10. For the formulations containing 300 mM C10, it was noted that the dextrans displayed an increased absorption rate (compared to 50 mM C10), and their absorption continued for at least 60 min. The absorption rate of MEDI7219, on the other hand, was similar at both studied C10 concentrations, but the duration of absorption was extended at the higher enhancer concentration, leading to an increase in the overall extent of absorption. The absorption of PEP12210 was similar in terms of the rate and duration at both studied C10 concentrations. This is likely caused by the instability of this molecule in the intestinal lumen. The degradation decreases the luminal concentrations over time, which in turn limits absorption at time points beyond 20 min. The results from this study show that permeation enhancement effects cannot be extrapolated between different types of macromolecules. Furthermore, to maximize the absorption of a macromolecule delivered together with C10, prolonging the duration of absorption appears to be important. In addition, the macromolecule needs to be stable enough in the intestinal lumen to take advantage of the prolonged absorption time window enabled by the permeation enhancer.

## Introduction

Oral administration of macromolecules remains of high interest in the drug delivery field to offer patients an alternative to injections. There has been some recent success using permeation enhancers with the approval of oral semaglutide and oral octreotide, but bioavailability remains low and variable.^1,2^ It is still not well understood how to best formulate permeation enhancers for oral administration of macromolecules. Sodium caprate (C10) is one of the most widely studied permeation enhancers and has been evaluated in several clinical studies.^3−5^ C10 is believed to work by enhancing both the paracellular and transcellular transport of co-presented macromolecules.^6−8^ We recently used the rat intestinal instillation model to better understand the absorption mechanism of C10.^9^ Higher C10 concentrations were correlated not only with higher fluorescein isothiocyanate (FITC)-dextran 4000 (FD4) bioavailability and *C*_max_ but also more erosion of the enterocyte layer. The epithelial erosion was transient; however, the enterocyte layer had recovered 120 min after administration. Our results suggest that at relevant *in vivo* concentrations, the mechanism of action seems to predominantly stem from a transient perturbation of the integrity of the intestinal epithelium. The current work extends on the previously published study to include a more diverse set of compounds. For this, a larger dextran, FITC-dextran 10 000 (FD10), a peptide (MEDI7219), and a protein (PEP12210), were selected and studied in the same rat intestinal instillation model.

MEDI7219 is a glucagon-like peptide 1 (GLP-1) receptor agonist drug candidate developed by AstraZeneca.^10^ The peptide was designed for oral delivery and has natural amino acid and α-methyl amino acid substitutions to protect against peptidasesin the gastrointestinal tract. The peptide backbone has two lipid side chains that can bind to plasma proteins, thereby prolonging the circulation half-life of the peptide. The half-life following oral administration to dogs was 9.8 h.^10^ The calculated pI is 3.8, giving the peptide a charge of −6.2 at pH 7.4. The molecular weight of MEDI7219 is 4353, i.e., comparable to FITC-dextran 4000. PEP12210 belongs to a class of affinity proteins known as affibody molecules, which are in development for both therapeutic and diagnostic purposes.^11−13^ PEP12210 consists of two covalently bound peptide domains, one target binding domain, and one albumin binding domain, designed to extend the plasma half-life to approximately two days in the rat. The target of PEP12210 is Taq polymerase, which is a DNA polymerase used in the polymerase chain reaction. PEP12210 is thus not pharmacologically active in humans or rats and is used here as a model of Affibody molecules. PEP12210 has a pI of 4.5 and carries 5 negative charges at pH 7.4. The molecular weight of PEP12210 is similar to FITC-dextran 10 000 at 11 740 Da. PEP12210 only contains natural amino acids and is thus more sensitive to enzymatic degradation in the gastrointestinal tract.

The aim of the study was to compare the absorption characteristics of FD10 to that of FD4 and to compare the absorption of MEDI7219 and PEP12210 to that of dextrans with similar molecular weights. Sodium caprate was chosen as permeation enhancer and studied at two different concentrations relevant for oral co-delivery with macromolecular drugs, with the objective to investigate its effect on the rate and extent of absorption of the macromolecules in the rat intestinal instillation model. The selected C10 concentrations were based on the assumption of an enteric-coated dosage form containing 500 mg of C10 (as in the case of GIPET I^14^) dissolving in the human small intestine. A concentration of 50 mM represents the case of drug release in the entire resting volume of the small intestine (typical volume 43–105 mL^15^). A concentration of 300 mM mimics the scenario where the entire dosage form dissolves in one intestinal fluid pocket (typical volume 4–12 mL^15^).

## Materials and Methods

### Materials

MEDI7219 was supplied by AstraZeneca and PEP12210 was supplied by Affibody. The other materials were purchased from the following sources: Fluorescein isothiocyanate-dextran 10 000, maleic acid, sodium phosphate dibasic anhydrous, monobasic potassium phosphate, Poloxamer 188 solution 10%, D-sorbitol, pancreatin (8X USP specifications), and sodium hydroxide from Sigma-Aldrich (St. Louis, MO); sodium caprate from Tokyo Chemical Industry (Tokyo, Japan); sodium chloride from Honeywell Fluka (Seelze, Germany); phosphate-buffered saline from Life Technologies Limited (Paisley, U.K.); glucose solution 5% for injection and NaCl 0.9% for injection from Braun (Melsungen, Germany); and 2 M HCl solution and 2 M NaOH solution from Apotek Produktion & Laboratorier (Gothenburg, Sweden). Water was purified with a Millipore Milli-Q Advantage A10 system (Millipore Corporation, Billerica, MA).

### Pancreatin Stability Study of PEP12210

The stability of PEP12210 to pancreatin was studied in a 50 mM phosphate buffer pH 6.8 containing 1 mg/mL pancreatin.^16^ In a study by Wang et al. where USP levels (10 mg/mL) of pancreatin were used, rapid degradation prevented half-life determination, especially for the linear peptides.^17^ As PEP12210 is a linear protein consisting of natural amino acids, a lower pancreatin level was selected to allow estimation of half-life. The incubations were performed on an Eppendorf Thermomixer comfort shaking incubator set to 37 °C and 1000 rpm. Monobasic potassium phosphate (6.8 g) was dissolved in 250 mL of water whereafter 77 mL of 0.2 M NaOH and 500 mL water were added, and pH was adjusted to 6.8 and volume to 1000 mL. A pancreatin stock solution was prepared by dissolving 25 mg of pancreatin (8X USP specifications) in 100 mL of phosphate buffer to give a concentration corresponding to 2 mg/mL (1X USP specifications). A stock solution of PEP12210 was prepared by dissolving the peptide at a concentration of 12 mg/mL in phosphate buffer. To start the study, 200 μL of preheated pancreatin stock solution was mixed with 200 μL of preheated PEP12210 stock solution. The digestion media thus contained 6 mg/mL of PEP12210 and 1 mg/mL pancreatin. After incubating for 5, 10, 15, 20, 30, 45, or 60 min, a 200 μL sample was taken and transferred to a pre-cooled vial containing 600 μL of 20 mM HCl to stop the digestion process. The content was mixed by pipetting and centrifuged at 10 000*g* for 10 min at +4 °C. The supernatant was transferred to a new vial, frozen on dry ice, and stored at −80 °C until analysis. The samples were separated using gel electrophoresis and quantified with SPYRO Ruby staining. 0.2 μg of peptide (prepared with 50 mM dithiothreitol (DTT) in LDS-sample buffer, Thermo Scientific) was loaded per lane on a 26-well, 4–12% NuPAGE Bis-Tris gel (Invitrogen). Sodium dodecyl sulfate-polyacrylamide gel electrophoresis (SDS-PAGE) was performed in pH 7.3 MES buffer at 200 V for 45 min. SYPRO Ruby protein gel stain (Invitrogen) was used according to manufacturer’s rapid protocol except that methanol was exchanged with ethanol. The gel was visualized using the Gel Doc EZ gel documentation system using the SYPRO Ruby intense band protocol and Image Lab software (Bio-Rad). Intensities of the bands were measured using the software. The intact peptide was noted as the fraction of the main band and additional bands that appeared below the main band were noted as a fraction of degraded peptide over time. The half-life was calculated using the following equation
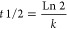
where *k* is the slope of the line formed when plotting the natural logarithm of the amount of intact peptide remaining versus time.

### Buffers and Formulations

Blank FaSSIF was prepared by dissolving 2.22 g of maleic acid, 1.39 g of sodium hydroxide, and 4.01 g of sodium chloride in Milli-Q water and adjusting the pH to 6.5 and volume to 1000 mL. C10 solutions were prepared at 50 or 300 mM C10 in blank FaSSIF and pH-adjusted to 6.5 by the addition of 2 M HCl. Blank FaSSIF solutions containing 0–300 mM C10 were stored at room temperature (RT) and used within 30 days. The model compounds were dissolved in blank FaSSIF with or without C10 the day before the absorption study and stored at +4 °C overnight. The following concentrations were used for the intestinal administrations: FD10: 12.5 mg/mL, MEDI7219 1.25 mg/mL, and PEP12210 6.25 mg/mL. The impact of including bile salts and phospholipids in the formulation was studied in our previous publication.^9^ No difference in the absorption of FD4 was observed when FD4 and C10 were prepared in the fasted and fed state simulated intestinal fluids FaSSIF-V2 or FeSSIF-V2, as compared to blank FaSSIF. Therefore, the simple buffer system (blank FaSSIF) was chosen for studying the absorption of macromolecules in this work. The formulation for IV administration of MEDI7219 was prepared in a 50 mM phosphate buffer containing 240 mM sorbitol, 0.02% poloxamer 188, and 0.1 mg/mL MEDI7219. The formulations for IV administration of FD10 and PEP12210 were prepared by dissolving the model compounds in pH 7.4 phosphate-buffered saline (PBS). FD10 was prepared at 3.0 mg/mL and PEP12210 at 0.6 mg/mL. All IV formulations were sterile filtered into autoclaved injection vials and stored at +4 °C.

### Rat Intestinal Instillation Model and IV Administration

The study was approved by the local ethics committee for animal research in Gothenburg, Sweden (ID 1995, approval 5 December 2018). Male Wistar Han rats (Charles River Laboratories, Germany), aged 10 to 14 weeks, with an average weight of 303 g (range 235–364 g, SD 32 g, CV 10.6%) were used. The animal housing room was maintained at 21 °C and 50% RH, with a 12 h light/dark cycle. Upon arrival at the animal facility, the rats underwent an acclimatization period of 5 days with food and water *ad libitum*. Prior to the experiment, the rats were fasted on grids for 16 h in separate cages with free access to water and a 5% glucose solution.

The absorption study was performed as previously described.^9^ Briefly, anesthesia was induced with isoflurane after which the animal was moved to a preheated preparatory table. Fur was shaved from the throat to the lower abdomen and the shaved area was disinfected with a medicinal sponge containing chlorhexidine. The rat was draped in plastic foil and transferred to a preheated operating table where anesthesia was continued throughout the entire study using 3% isoflurane carried in an air/oxygen mixture. A polyurethane catheter was placed in the left carotid artery to allow for blood sampling, as well as blood pressure and heart rate monitoring. A midline incision was made in the abdomen, the duodenum was located and a catheter was placed in the common bile duct to avoid bile secretion into the duodenum. We studied the impact of bile salts and phospholipids in our previous publication by preparing formulations in simple buffer, FaSSIF-V2 or FeSSIF-V2.^9^ No influence was observed in this experiment. However, since bile duct catheters were used in the previous study on FD4, we have chosen to keep the experimental setup the same when studying the macromolecules of the current work. The stomach was punctured approximately 1 cm proximal to the pyloric sphincter with a 20G needle. A soft polyurethane catheter with a rounded tip was inserted into the small intestine via the gastric incision so that the tip of the catheter was positioned approximately 4 cm distal to the pylorus. The intestinal catheter was secured to the stomach with a suture and a ligature was placed at the pylorus to prevent backflow of formulation into the stomach and transit of gastric content into the duodenum. A thermometer was placed in the abdominal cavity and the abdomen was closed with stitches. A heating lamp coupled to the thermometer via a thermostat maintained the animal at 37 °C. The surgery was followed by a 30 min recovery period to allow the animal to regain normal temperature and blood pressure.

The liquid formulations were equilibrated to room temperature under magnetic stirring for 2 h prior to administration. Intestinal bolus administrations were performed over approximately 5 s via the intestinal catheter. The dose volume was 0.8 mL in all cases resulting in the following total doses FD10: 10 mg, MEDI7219: 1.0 mg, and PEP12210: 5.0 mg. The C10 doses were 7.8 mg for administrations with 50 mM C10 and 47 mg for 300 mM C10. Five to six replicates were performed for each administration group. Blood samples were drawn before administration and at 5, 10, 20, 30, 45, 60, and 120 min post-administration. Blood plasma was separated by immediately centrifuging the blood samples at +4 °C and 10 000*g* for 4 min. Plasma samples were transferred to new tubes and stored at −80 °C until analysis. Animals that did not maintain an average blood pressure of 70 mmHg or higher during the administration and blood sampling period were excluded to secure normal physiological conditions.

The animals receiving IV administration were handled and prepared in the same way as the animals receiving intestinal administration up until the placement of the carotid catheter. As no abdominal surgery was performed, a thermometer was introduced rectally. A recovery period of 30 min was allowed before IV administration into the tail vein. The following dose levels were used: FD10: 5 mg/kg, MEDI7219: 0.05 mg/kg, and PEP12210: 1 mg/kg.

### Bioanalysis of Plasma Samples

#### FITC-Dextran 10 000 Quantification from Plasma

The plasma samples were thawed and 80 μL of plasma was transferred to a 96-well plate (Thermo Fisher Scientific, Waltham). For quantification, two sets of calibration standards with known concentrations of FD10 in blank plasma were added to the 96-well plate. Stock solutions of FD10 were prepared in PBS, pH 7.4, and stored at −80 °C. Calibration samples were prepared by a 20-fold dilution of stock solutions in species-matched blank plasma. The plates were analyzed for fluorescence emission using a Multimode plate reader (PerkinElmer, Waltham), with excitation at λ 494 nm and emission at λ 518 nm. Study samples were quantified with four parameter logistic regression against the response from the calibration standards.

#### MEDI7219 Quantification from Plasma

Plasma samples were prepared by protein precipitation with an organic solvent. Cold acetonitrile/methanol (1:1, 180 μL) with 0.2% formic acid and 10 nM internal standard (5,5-diethyl-1,3-diphenyl-2-iminobarbituric acid) was added to 50 μL of plasma. Samples were vortexed for 1 min and centrifuged at 4000*g* for 20 min at 4 °C. The supernatant was transferred to a Protein LoBind microplate (Eppendorf, Hamburg Germany) and diluted 1:1 with 0.2% formic acid in purified water, and analyzed with liquid chromatography–mass spectrometry (LC–MS)/MS. Further dilution was made with acetonitrile/methanol/water (1:1:2) and 0.2% formic acid, if needed. Calibration standards were prepared in species-matched blank plasma using Protein LoBind Eppendorf tubes. All sample preparation was performed on wet ice. The LLOQ for MEDI7219 was 1.06 nM. Standard curve samples ranged from approximately 1 nM to 40 μM. The accuracy and precision for all calibration standards were within ±20% of nominal concentration. Samples were analyzed on an ACQUITY Premier system coupled to a Xevo TQ-XS triple quadrupole mass spectrometer (Waters Corporation, Milford, MA). MEDI7219 was separated on an Acquity UPLC peptide BEH C18 column (2.1 × 50 mm, with 1.7 μm particle size and 300 Å pore size). Mobile phase A was 0.2% formic acid and 2% acetonitrile in purified water. Mobile phase B was 0.2% formic acid in acetonitrile. The compounds were separated with a linear gradient from 4 to 80% B from 0.0 to 2.5 min and then 80–95% B from 2.5 to 2.6 min, 95% B from 2.6 to 3.3 min, and 95–4% B from 3.3 to 3.4 min, and then two cycling steps of 4–95% B and back to 4% B in 0.6 min. The flow rate was 0.6 mL/min and the column temperature was 50 °C. The sample manager and sample organizer temperature were set to 11 °C. The compounds were analyzed in positive mode using electrospray ionization. MRM settings used: MEDI7219 [M + 4H]^4+^*m*/*z* 1089.01 > 1084.68 (cone voltage: 60 V, collision energy: 18 V); internal standard [M + H]^+^*m*/*z* 336.11 > 194.92 (cone voltage: 22 V, collision energy: 34 V). Study samples were quantified with linear regression against the response from the calibration standards with 1/X2 weighting. The response was calculated as the peak area of the analyte divided by the peak area of the internal standard.

#### PEP12210 Quantification from Plasma

PEP12210 was quantified from the plasma using an indirect sandwich enzyme-linked immunosorbent assay (ELISA). Mouse anti-Z monoclonal antibody (2 μg/mL) in PBS (50 μL/well) were coated on 96-well half area plates and incubated overnight at 4 °C. The plates were washed with 0.5% Tween 20 in PBS (PBST) and blocked with BlockerCasein (Thermo Scientific cat. no. 37528) for 1.5 h at 22 °C. The PEP12210 standard material was titrated in a 1.5-fold dilution series (5–200 pM) in 1% rat plasma pool in BlockerCasein. Plasma samples were diluted 1:100 in BlockerCasein followed by 1:3 serial dilution in 1% rat plasma pool. Plasma samples from IV dosed animals were subjected to an additional 1:100 dilution in 1% rat plasma pool before serial dilution. Calibration standards and diluted plasma samples were added to coated ELISA plates (50 μL/well) and incubated for 1.5 h at 22 °C. Following washing with PBST, a rabbit anti-ABD polyclonal antibody (2 μg/mL) was added. After incubation for 1.5 h at 22 °C, the plates were washed with PBST and 100 ng/mL horseradish peroxidase-conjugated donkey anti-rabbit IgG (Jackson Immuno Research cat. no. 711-035-152) was added to each well. After one additional hour of incubation and subsequent washing in PBST, the plates were developed with 3,3′,5,5′-tetramethylbenzidine (TMB) (50 μL/well) for 15 min at RT, and the reactions were stopped by addition of 0.2 M H_2_SO_4_ (50 μL/well). The absorbance at 450 nm was measured in a microplate plate reader (PerkinElmer Enspire LF). The LLOQ of the assay was 0.8 nM.

#### Pharmacokinetic Analysis

The plasma concentration versus time data were analyzed using non-compartmental analysis using Phoenix WinNonlin version 8.2 (Certara USA, Inc., Princeton, NJ). The area under the individual plasma concentration–time curve (AUC) from time zero to 120 min (AUC_0-120_), was calculated with the linear trapezoidal method. Bioavailability from zero to 120 min after intraduodenal administration was calculated according to
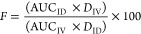
where *F* is the bioavailability in percent, AUC is the area under the plasma concentration–time curve from time zero to 120 min, *D* is the administered dose, intraduodenally (ID) or intravenously (IV). Numerical deconvolution was used to estimate the cumulative fraction absorbed over time as described by Langenbucher.^18^ The average, dose-normalized plasma concentrations following intravenous administration of the compounds were used as the weighting function and dose-normalized plasma concentrations following duodenal administrations were assigned to the response function. The deconvolution was carried out using GI-Sim^19−21^ version 5.4 with the time step set to 0.5 min. The mean cumulative input function for each administration group is plotted which corresponds to the fraction absorbed.

The FD4 data are replicated from a previous study performed by our group using the same methodology. The FD4 formulation was prepared in blank FaSSIF, pH 6.5, containing 0–300 mM C10 and 12.5 mg/mL FD4, and was administered as a 0.8 mL bolus, giving a dose of 10 mg of FD4.^9^

#### Statistical Analysis

Statistical analysis was performed using R (version 3.6.3, R Foundation for Statistical Computing, Vienna, Austria). A two-way analysis of variance (ANOVA) was used to compare the bioavailability values for the compounds at each C10 concentration, to account for multiple comparisons, *p*-values were adjusted using the Sidak method. The following compound comparisons were performed: PEP12210–FD10, MEDI7219–FD4, and FD4–FD10. Statistical significance was declared for *p* < 0.05.

## Results

### Enzymatic Stability of the Macromolecules

**Figure 1:**
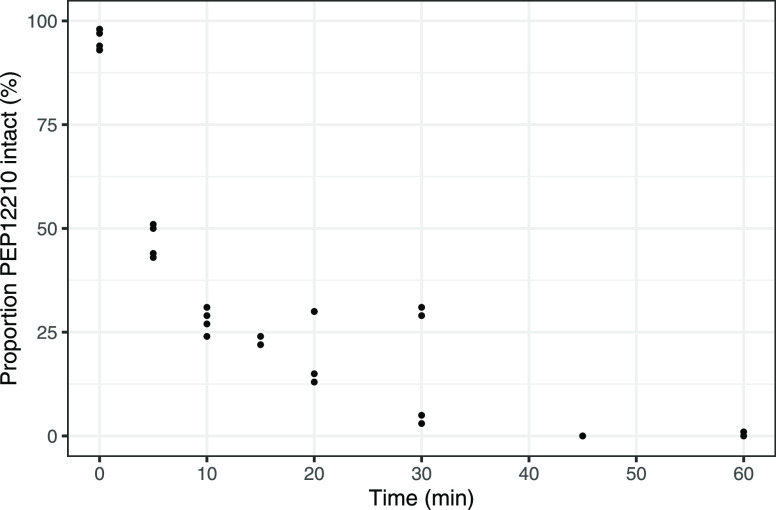
*In vitro* digestion of PEP12210. Proportion of intact PEP12210 remaining versus time after incubation at 6 mg/mL together with 1 mg/mL pancreatin in phosphate buffer pH 6.8. *n* = 2–4 per timepoint. Each point is a separate incubation.

The stability of PEP12210 when digested with pancreatin is shown in Figure 1. Approximately 50% of PEP12210 was degraded already at 5 min, at 10 min 28% of the protein remained intact and by 45 min no intact protein could be detected. The half-life of PEP12210 was estimated to be 6 min. The stability of MEDI7219 in simulated intestinal fluid containing pancreatin was inferred from the published literature, showing that 90% of the peptide remained intact over a 60 min period.^10^ In a study by Mehvar and Shepard, FITC-dextrans were administered orally to rats and the excreted FITC-dextrans were analyzed from urine.^22^ The molecular weights of the excreted FITC-dextran 4000 and FITC-dextran 20 000 were similar to that of the administered dextrans, indicating that these FITC-dextrans are not degraded to any significant extent in the gastrointestinal tract.

### Intestinal Absorption of the Different Macromolecules

**Figure 2:**
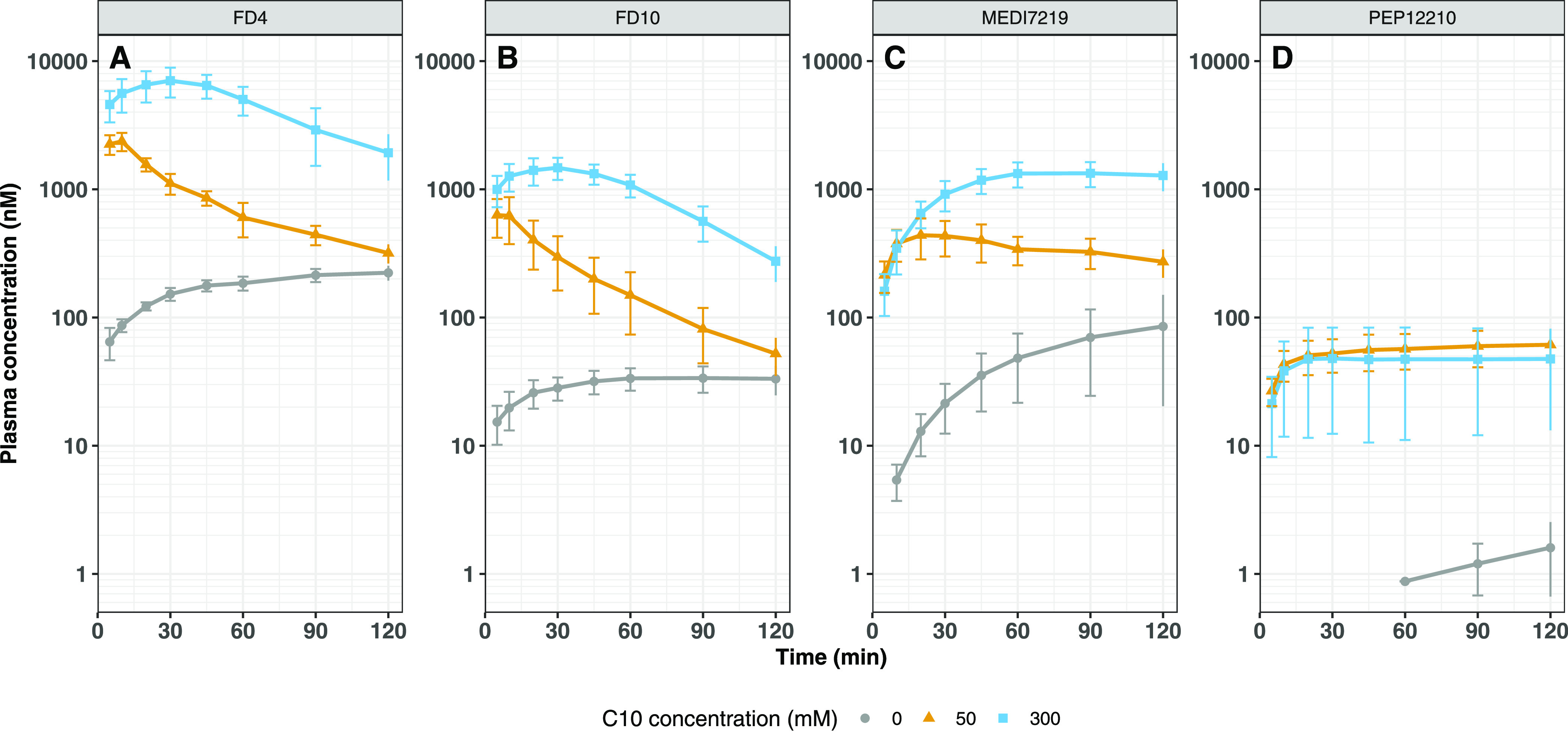
Plasma concentration–time profiles following intestinal administration to anesthetized rats. FD4 (A), FD10 (B), MEDI7219 (C) and PEP12210 (D). Formulations contained 0 (gray circles), 50 (yellow triangles) or 300 mM C10 (blue squares) in maleate buffer adjusted to pH 6.5. Average of 5–6 animals per group, error bars indicate ±SD. FD4 data is replicated from ref (9).

The plasma concentration–time profiles for the studied compounds are shown in Figure 2 and the corresponding pharmacokinetic parameters are presented in Table 1. The bioavailability of FD10 was approximately two times lower than that of the smaller dextran, FD4, whether studied in the presence or absence of C10 (*p* < 0.05 for all groups). The bioavailability of MEDI7219 was 2–4 times lower compared to FD4 across all groups, despite having a similar molecular weight (*p* < 0.001 for all groups). Intestinal absorption of PEP12210 was negligible in the absence of C10, only two animals displayed plasma levels above the lower limit of quantification and only at later time points. When C10 was included in the formulations, complete pharmacokinetic profiles were also obtained for PEP12210. The bioavailability of PEP12210 was 20-fold or more lower compared to FD10, both for the control groups lacking C10 and for the administration groups including 50 or 300 mM C10 (*p* < 0.0001 for all groups).

**Table 1:** Pharmacokinetic Parameters for the Four Macromolecules Following Intestinal Administration to Anesthetized Rats^a^

compound	C10 conc. (mM)	*F* (%)	enhancement ratio	*C*_max_ (nM)	*T*_max_ (min)
**FD4**	0	1.6 ± 0.15 (9.2)	1 ± 0.092 (9.2)	230 ± 30 (13)	90 (90–120)
**FD4**	50	7.9 ± 1.2 (15)	4.9 ± 0.72 (15)	2400 ± 400 (17)	10 (5–10)
**FD4**	300	44 ± 11 (26)	27 ± 7.1 (26)	7100 ± 1700 (23)	30 (30–45)
**FD10**	0	0.56 ± 0.11 (20)	1 ± 0.2 (20)	35 ± 6.5 (18)	120 (45–120)
**FD10**	50	4.0 ± 1.7 (42)	7.1 ± 3.0 (42)	650 ± 230 (36)	5 (5–10)
**FD10**	300	18 ± 3.2 (18)	31 ± 5.7 (18)	1500 ± 280 (18)	30 (10–30)
**MEDI7219**	0	0.41 ± 0.25 (62)	1 ± 0.62 (62)	85 ± 65 (76)	120 (120–120)
**MEDI7219**	50	3.1 ± 0.85 (28)	7.5 ± 2.1 (28)	440 ± 140 (32)	20 (20–30)
**MEDI7219**	300	9.5 ± 1.9 (20)	23 ± 4.7 (20)	1400 ± 320 (23)	90 (60–120)
PEP12210*	0	0.0021 ± 0.00074 (36)	1 ± 0.36 (36)	1.6 ± 0.94 (58)	120 (120–120)
**PEP12210**	50	0.17 ± 0.052 (31)	82 ± 25 (31)	61 ± 20 (32)	120 (120–120)
**PEP12210**	300	0.14 ± 0.10 (73)	67 ± 49 (73)	50 ± 35 (71)	120 (120–120)

a *n* = 5–6 per group. Values are given as mean ± SD (CV%), except *T*_max_, which is given as median (min–max). FD4 data from ref (9). *n = 2 due to most levels below LLOQ.

### Absorption–Time Profiles of the Different Macromolecules

**Figure 3:**
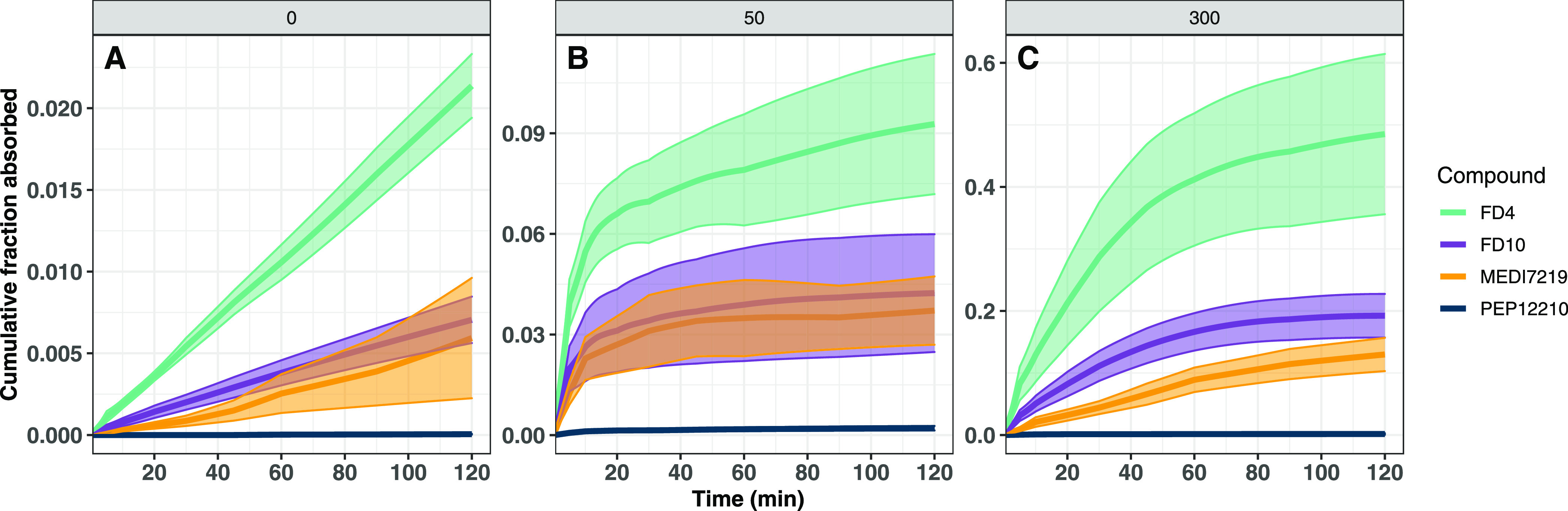
Cumulative fraction absorbed versus time contrasted for the four studied macromolecules. Formulations contained 0 (A), 50 (B), or 300 mM C10 (C) in maleate buffer adjusted to pH 6.5, *n* = 5–6 per group. Line shows the average value and the shaded area indicates ±SD. FD4 data are taken from ref (9).

**Figure 4:**
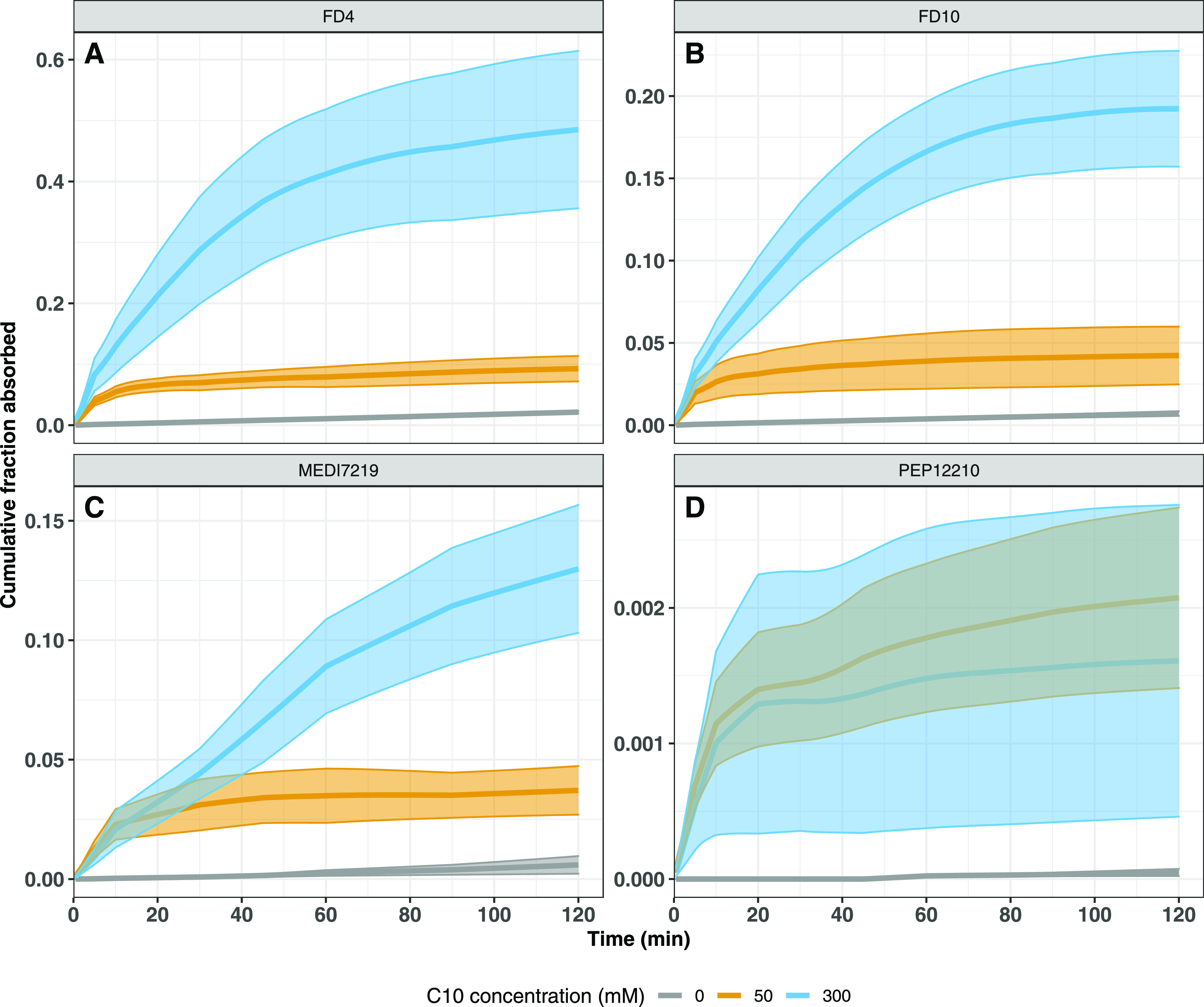
Effect of the concentration of the permeation enhancer on the cumulative fraction absorbed for each compound separately. The plots show cumulative fraction absorbed for FD4 (A), FD10 (B), MEDI7219 (C), and PEP12210 (D). Formulations contained 0 (gray), 50 (yellow), or 300 mM C10 (blue) in maleate buffer adjusted to pH 6.5, *n* = 5–6 per group. Line shows the average value and shaded area indicates ±SD. FD4 data are taken from ref (9).

The absorption profiles over time for the studied macromolecules are presented in Figures 3 and 4. Table 2 lists the proportion of the total absorption taking place in selected time intervals for each group separately. In the absence of C10, absorption rates for all four macromolecules, albeit low, were constant over the time period of investigation (Figure 3A). When 50 mM C10 was included in the formulations, the rate of absorption for all molecules was increased at early time points before returning to similar absorption rates as observed in the absence of C10 (Figure 3B and Table 2). When the C10 concentration in the formulation was increased to 300 mM, absorption rates further increased but only for FD4 and FD10 (Figures 3C and 4A,B). Interestingly, and unlike for the FITC-dextrans, no increase in the rate of absorption of MEDI7219 was observed with increasing C10 concentration, rather only the duration of enhanced absorption was prolonged (Figure 4C). For PEP12210, inclusion of C10 in the formulation increased the absorption rate at early time points only and no difference was observed in the duration of absorption when comparing 50 and 300 mM C10 formulations, with absorption diminishing after approximately 20 min (Figure 4D).

**Table 2:** Proportion of Total Absorption Over the Time Course of Study Occurring in Selected Intervals Following Intestinal Administration to Anesthetized Rats^a^

		time interval					
compound	C10 conc. (mM)	0–20 min	20–40 min	40–60 min	60–80 min	80–100 min	100–120 min
**FD4**	0	17% ± 1.5%	17% ± 1.6%	16% ± 1.0%	16% ± 0.79%	17% ± 1.1%	17% ± 2.3%
**FD4**	50	72% ± 6.8%	8.3% ± 3.8%	5.1% ± 2.8%	5.7% ± 1.5%	5.2% ± 1.5%	3.7% ± 0.19%
**FD4**	300	44% ± 8.5%	27% ± 1.7%	15% ± 3.7%	7.3% ± 3.3%	3.9% ± 3.1%	3.5% ± 1.1%
**FD10**	0	20% ± 4.1%	17% ± 1.1%	17% ± 0.83%	16% ± 1.3%	15% ± 2.2%	15% ± 2.1%
**FD10**	50	74% ± 7.1%	12% ± 2.3%	5.8% ± 1.8%	4.0% ± 1.5%	2.4% ± 1.7%	2.2% ± 2.0%
**FD10**	300	43% ± 8.7%	27% ± 1.2%	17% ± 3.4%	8.6% ± 2.8%	3.5% ± 2.2%	1.2% ± 0.76%
**MEDI7219**	0	11% ± 2.8%	12% ± 2.9%	22% ± 2.5%	15% ± 1.2%	18% ± 2.5%	22% ± 4.9%
**MEDI7219**	50	72% ± 3.6%	16% ± 2.6%	4.5% ± 1.7%	1.8% ± 5.5%	2.1% ± 2.6%	3.6% ± 4.2%
**MEDI7219**	300	25% ± 5.1%	20% ± 2.6%	24% ± 2.3%	13% ± 3.5%	10% ± 3.1%	7.8% ± 4.0%
PEP12210*	0	0% ± 0%	0% ± 0%	56% ± 31%	9.9% ± 14%	17% ± 8.6%	17% ± 8.5%
**PEP12210**	50	68% ± 5.9%	7.0% ± 4.4%	11% ± 1.6%	6.1% ± 0.93%	4.9% ± 0.44%	3.0% ± 0.62%
**PEP12210**	300	79% ± 9.6%	2.5% ± 3.5%	9.3% ± 2.0%	4.0% ± 1.9%	3.3% ± 1.6%	2.2% ± 1.6%

a Values given as average proportion (%) ±SD of the total fraction absorbed during the 120 min investigative period. The total of every row adds up to 100%. *n* = 5–6 per group, except * where *n* = 2. FD4 data are taken from ref (9).

## Discussion

In agreement with our previous study on FD4, the rate and duration of absorption of FD10 increased with increasing C10 concentration. As discussed in our previous paper, this seems to correlate well with the time-dependent histological changes of the intestinal epithelium observed following intestinal administration of C10 at different concentrations.^9,23^ Furthermore, the bioavailability of FD10 was approximately 2-fold lower than that of FD4, consistent with the previously published data where these two dextrans have been co-delivered with other permeation enhancers and studied in rodent models.^24,25^ These data further confirm the dependence of absorption on the molecular weight of the macromolecule when co-delivered with a permeation enhancer.

The rate and extent of absorption of MEDI7219 were observed to be lower than FD4 even if both molecules have similar molecular weights, and more resembled the absorption of FD10 (Figure 3 and Table 1). Further examination of the absorption–time profiles reveals that MEDI7219 displays a similar absorption rate when delivered together with both 50 and 300 mM C10 (Figure 4C). This implies that the increased bioavailability of MEDI7219 seen when delivered with 300 mM C10 mainly stems from a prolonged duration of absorption, not from an increased absorption rate. This is in contrast to the two FITC-dextrans, for which both an increased absorption rate and duration were observed (Figure 4A,B). This suggests that for FITC-dextrans, higher luminal C10 concentrations will result in a greater extent of absorption, while also suggesting that for MEDI7219, maintaining a lower luminal C10 concentration for a prolonged time period may result in the same total extent of absorption as delivering a higher initial C10 concentration as a bolus. Maintaining a lower C10 concentration over a prolonged time period could improve the safety of the drug delivery system as the lower C10 concentration would be expected to have less impact on the intestinal epithelium as reported in our previous study.^9^ However, the benefits of a prolonged release of permeation enhancer are not supported by Tyagi et al., where formulations with a slower, more prolonged release did not show any benefits in improving the bioavailability of MED7219.^26^ It should be noted, however, that different permeation enhancers (a combination of sodium chenodeoxycholate and propyl gallate) were used in the study by Tyagi et al.,^26^ which could also impact the outcome of the study.

PEP12210 was absorbed to a much lower extent compared to the other compounds (Table 1 and Figure 3). Similar to the other three compounds, the majority of PEP12210 absorption took place during the first 20 min after administration in the presence of 50 mM C10 (Figure 4D and Table 2). Interestingly, increasing the C10 concentration of the formulation from 50 to 300 mM did not result in an increased rate nor extended the duration of absorption (Figure 4D). One reason for the contrasting behavior in the duration of absorption is likely to be found in the enzymatic instability of this molecule (Figure 1). The half-life of PEP12210 in the presence of pancreatin was approximately 6 min. Due to the expected rapid degradation in the intestinal lumen, the concentration gradient across the epithelium would be expected to quickly drop, decreasing the driving force for absorption via passive pathways. For administrations with a higher C10 concentration (300 mM), no additional absorption was seen beyond 20 min. By this timepoint, little intact protein is expected to be left in the intestinal lumen. Therefore, even if the intestinal epithelium still displays an increased permeability due to exposure to 300 mM C10, no continued absorption of PEP12210 is observed.

Overall, and acknowledging the limited sample set of macromolecules included, this study shows that molecular weight and enzymatic stability are two important factors that govern the oral bioavailability of macromolecules when formulated together with permeation enhancers. In terms of enzymatic stability, and based on the absorption profiles where increased rates of absorption are observed over 30–60 min post-administration (depending on the concentration of C10 in the formulation), then it would seem reasonable to assume that adequate stability over this time period would be required to fully exploit the permeation enhancing effects afforded by the permeation enhancer. Our results further indicate that molecular weight and enzymatic stability are not the only determinants of the extent of absorption, and that other physicochemical properties of the macromolecule play a role, particularly when it comes to the effect of the permeation enhancer on the rate of absorption. This may have implications in terms of how to optimize the design of oral dosage forms combining macromolecules with permeation enhancers and highlights the need for more research into the molecular properties of macromolecules that affect absorption following oral administration together with permeation enhancers.

## Conclusions

The bioavailability of FD4 was approximately twice that of FD10, confirming that molecular weight is an important factor when using permeation enhancers for improving the intestinal absorption of macromolecules. However, absorption enhancing effects of permeation enhancers cannot be explained by molecular weight alone and cannot be extrapolated between different types of macromolecules having similar molecular weight as shown by the different results obtained for the studied macromolecules under well-controlled *in vivo* conditions. Differences in chemical and enzymatic stability, as well as physicochemical properties, are likely of importance for the absorption and systemic availability of the macromolecule, also when delivered together with permeation enhancers. Our results illustrate that for proteolytically stable macromolecules, the time window during which the macromolecule maintains an increased permeability through the intestinal epithelium, is dependent on the amount of C10 presented to the epithelium. Thus, the increased absorption observed by increasing the amount of permeation enhancer is not only a direct effect on permeability but also related to the increased duration of permeation enhancement. Whether this can also be achieved by sustaining the release of C10 to maintain lower concentrations over a longer period of time, remains to be verified. However, to take full advantage of an extended absorption time window enabled by the permeation enhancer, the macromolecule needs adequate stability in the gastrointestinal tract.
